# The Peripheral Stalk of Rotary ATPases

**DOI:** 10.3389/fphys.2018.01243

**Published:** 2018-09-04

**Authors:** Lilia Colina-Tenorio, Alain Dautant, Héctor Miranda-Astudillo, Marie-France Giraud, Diego González-Halphen

**Affiliations:** ^1^Departamento de Genética Molecular, Instituto de Fisiología Celular, Universidad Nacional Autónoma de México, Mexico City, Mexico; ^2^CNRS, UMR5095, IBGC, Bordeaux, France; ^3^Energy Transducing Systems and Mitochondrial Morphology, Université de Bordeaux, Bordeaux, France; ^4^Genetics and Physiology of Microalgae, InBios, PhytoSYSTEMS, University of Liège, Liège, Belgium

**Keywords:** peripheral stalk, ATP synthase, coiled-coils, archaea, bacteria, mitochondria, chloroplast

## Abstract

Rotary ATPases are a family of enzymes that are thought of as molecular nanomotors and are classified in three types: F, A, and V-type ATPases. Two members (F and A-type) can synthesize and hydrolyze ATP, depending on the energetic needs of the cell, while the V-type enzyme exhibits only a hydrolytic activity. The overall architecture of all these enzymes is conserved and three main sectors are distinguished: a catalytic core, a rotor and a stator or peripheral stalk. The peripheral stalks of the A and V-types are highly conserved in both structure and function, however, the F-type peripheral stalks have divergent structures. Furthermore, the peripheral stalk has other roles beyond its stator function, as evidenced by several biochemical and recent structural studies. This review describes the information regarding the organization of the peripheral stalk components of F, A, and V-ATPases, highlighting the key differences between the studied enzymes, as well as the different processes in which the structure is involved.

## Introduction

ATP, a key molecule synthesized by ATP synthases, is instrumental for the metabolism of every living organism (Müller and Grüber, 2003). ATP synthases belong to a family of enzymes known as rotary ATP synthases, which are multiprotein enzymatic complexes embedded in cellular and organellar membranes of all organisms across the three life domains. These enzymes work as nanomotors to synthesize or hydrolyze ATP, and they have been classified in three types: F, V, and A-type ATPases. It should be acknowledged that there is another type of rotary ATPases that are Na^+^-selective and have been named N-type ATPases (Dibrova et al., 2010), however, these enzymes will not be discussed in this review.

F-type ATPases are found in the bacterial plasma membrane, in the inner mitochondrial membrane and in the thylakoid membrane of chloroplasts. F-type enzymes use an electrochemical proton gradient to synthesize ATP, according to the basic mechanism proposed by Mitchell (1961) in his chemiosmotic theory. These enzymes, in certain physiological conditions, can function in reverse and hydrolyze ATP to restore the membrane potential (D’Alessandro and Melandri, 2010). V-type ATPases were first purified from vacuoles, hence their name, and work as proton pumps dependent on ATP hydrolysis, which is why they are also known as H^+^-ATPases (Forgac, 2007). A-type ATPases are found in archaea and can function either synthesizing or hydrolyzing ATP (Grüber et al., 2014). All rotary ATPases have an ion channel in contact with a central stalk (or rotor) whose movement induces the conformational changes in the catalytic subunits of the hydrophilic domain that lead to synthesis/hydrolysis of ATP; and they all have the capacity to mechanochemically couple a rotary membrane domain (the proton channel) with a hydrophilic catalytic domain (Soga et al., 2017). Among other things, this coupling (pairing) is possible because of the structure known as peripheral arm or peripheral stalk, which works as the stator of a motor and whose main role is to counteract the rotation tendency of the catalytic core that happens in response to the movement of the rotor (Walker and Dickson, 2006).

The most widely accepted hypothesis about the origin of rotary ATPases states that they evolved from a common ancestor, which gave rise to the three types of enzymes. Initially, it was proposed that they had at least two transitions in their evolutionary history: the first was the transition from a proton pump to an ATP synthase driven by protons, and the second was the return to a proton pump (Cross and Taiz, 1990). Later, a third transition back to an ATP synthase was proposed, in which there was a gain in function, unlike the first two transitions (Cross and Müller, 2004). It is currently considered that the last universal common ancestor (LUCA) was in all likelihood a chemiosmotic organism with an ATP synthase in its membrane (Mulkidjanian et al., 2007). A-type ATPases are more closely related to V-type ATPases, although the latter cannot synthesize ATP in physiological conditions; it can thus be said that A-type ATPases are more similar to F-type ATPases in terms of mechanism (Forgac, 2007). Given the common origin of A and V-type ATPases, their catalytic subunits and their rotor subunits share 50% identity, while A and F-type ATPases share 25% identity (Müller and Grüber, 2003). On the contrary, the subunits of the peripheral stalk of F-ATPases are considerably less conserved, vary from one organism to the next, and no significant identity has been found among them (Muench et al., 2011).

**FIGURE 1:**
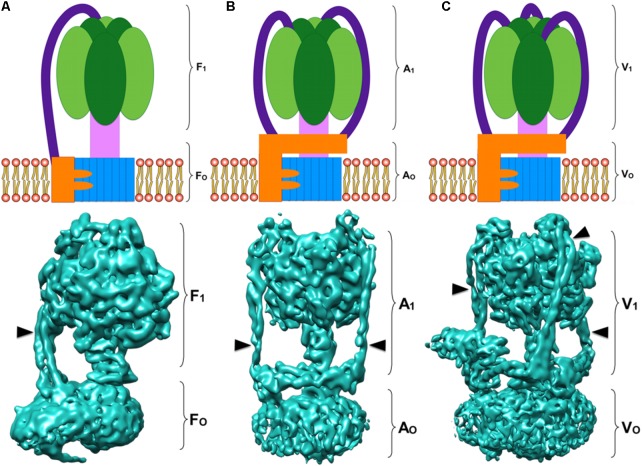
Rotary ATPases. **(A)** Schematic representation of an F-ATPase (Top) and three dimensional structure of the bovine heart mitochondria F-ATPase (Bottom) (Zhou et al., 2015) EMD 3164. **(B)** Schematic representation of an A-ATPase (Top) and three dimensional structure of the *Thermus thermophilus* A-ATPase (Bottom) (Schep et al., 2016) EMD 5335. **(C)** Schematic representation of a V-ATPase (Top) and three dimensional structure of the *Saccharomyces cerevisiae* V-ATPase (Bottom) (Zhao et al., 2015) EMD 6285. All the three dimensional maps were generated from electron cryo-microscopy images. The colors in the schematizations represent: catalytic core in dark and light green, central rotor in light pink, *c*-ring oligomer in blue, subunit *a* in orange, and peripheral stalks in dark purple. The arrowheads point to the peripheral stalk(s).

In terms of structure, the three types of ATPases are built in a similar way: a membrane domain (classically known as F_O_, V_O_, A_O_, or R_O_ to refer to this domain in general) that includes the proton channel and one or more peripheral stalks; and a soluble domain (classically known as F_1_, V_1_, A_1_, or R_1_ to refer to this domain in general) that includes: the catalytic domain (three pairs of catalytic subunits) and the central stalk, which communicates the activity of the proton channel with the catalytic subunits (Qi et al., 2007; Wächter et al., 2011; Grüber et al., 2014). The number of peripheral stalks has been used to categorize rotary ATPases (Stewart et al., 2013): F-ATPases have one (**Figure 1A**), A-ATPases have two (**Figure 1B**), and V-ATPases have three (**Figure 1C**). Although peripheral stalks have a similar function in all the enzymes, their composition and topology vary.

## The Peripheral Stalk

### The Structure of the Peripheral Stalk

As stated above, the peripheral stalk of rotary ATPases works as a stator and mediates the association of the membrane domain and the soluble domain of the enzyme. It is the most divergent component in both sequence and subunit composition, and three main roles have been attributed to this structure throughout its study, these will be discussed later. There are currently high resolution structures of the three types of rotary ATPases from model organisms: bovine (Zhou et al., 2015), yeast (Zhao et al., 2015; Hahn et al., 2016; Guo et al., 2017; Srivastava et al., 2018), and bacteria (Morales-Rios et al., 2015; Sobti et al., 2016), and of their peripheral stalks (Dickson et al., 2006; Oot et al., 2012; Stewart et al., 2012). These structures have shown that, in spite of the lack of sequence homology, the overall architecture of the peripheral stalk is similar in these enzymes.

The proteins that construct the peripheral stalk of rotary ATPases have to cover a distance of more than 100 Å from the membrane to the apex of the enzyme, and in order to achieve this most of these proteins have adopted long coiled coil structures (Stewart et al., 2013). Coiled coils are a common structural arrangement that is usually adopted by helical proteins, both fibrous and globular, and results from a particular alternate pattern of hydrophobic and hydrophilic amino acid residues in the sequence of the protein (Lupas, 1996). Three characteristics distinguish coiled coils from other amphipathic helices: (i) the periodicity of the hydrophobic residues (3.5 in coiled coils, 3.65 in other helices), (ii) the length of the helices (Su et al., 1994), and (iii) the packing interactions of the lateral chains. In coiled coils, distinctively, each residue of one helix fits in a space surrounded by two or four residues of the adjacent helix. This type of packing has been called “knobs into holes” or “in register packing” (Lupas, 1996). The amino acids of the pattern that gives rise to coiled coils are essential to maintain the structure of individual helices (through intramolecular interactions), as well as to promote specific interactions between more helices (through intermolecular interactions) (Mason and Arndt, 2004).

When the coiling of the helices is left-handed, it is the result of repetitive motifs in the sequence of the protein known as “heptad repeats.” Heptad repeats are a seven-residue pattern with an *abcdefg* composition, in which *ad* correspond to hydrophobic residues and e.g., to charged residues (Lupas, 1996). The nature of the residues in positions *ad*, as well as their equivalents in longer patterns, determines the number of chains involved in the formation of one functional unit of coiled coils, as was revealed by the study of the leucine zipper of the yeast transcription factor GCN4 (O’Shea et al., 1991). When the coiling of the helices is right-handed, it can be the result of either “hendecad repeat” motifs, which are eleven-residue patterns with an *abcdefghijk* composition, in which *adeh* correspond to hydrophobic residues; or of quindecad repeats (Stewart et al., 2013).

The first report of hendecad repeat patterns in an ATP synthase was of the subunits of the peripheral stalk of the F-ATPase from *Escherichia coli*, along with the prediction of a right-handed coiling of their helices (Del Rizzo et al., 2002). This type of coiling was later found in the peripheral stalks of A and V-ATPases (Stewart et al., 2013, 2014). The peripheral stalk subunits of mitochondrial F-ATPases show no canonical right-handed motifs in their sequence, but despite this, the structure in the yeast and mammalian enzymes is constructed by proteins with a helical conformation and maintains features reminiscent of the A and V-type ATPases overall architecture (Stewart et al., 2012), as will become evident in the following sections. An interesting insight into the functional implications of the right-handed coiling of the helices of the peripheral stalk was provided by Stewart et al. (2014), who studied the peripheral stalks of the *Thermus thermophilus* A-ATPase and found this direction of coiling results in an arrangement that makes them rigid in the direction of rotation. The authors discuss that this observation is consistent with the evolution of this protein fold to oppose the twisting force (an average torque value of ≈50 pN⋅nm; Omote et al., 1999) of the enzyme while also allowing some flexibility in the perpendicular direction to accommodate the conformational changes of the catalytic core subunits (Stewart et al., 2014).

### The Peripheral Stalk of F-ATPases

As previously stated, the peripheral stalk of F-ATPases varies considerably in its subunit composition and topology, from two subunits in *E. coli* (Dunn et al., 2000) to nine subunits in organisms like *Polytomella* sp. (Vázquez-Acevedo et al., 2006; Cano-Estrada et al., 2010). Within this variety, the most studied enzymes are those from bacteria, yeast, and mammals, however, the available evidence regarding the enzymes from non-model organisms shows they display interesting and highly divergent features.

#### The Peripheral Stalk of the Bacterial F-ATPase

**FIGURE 2:**
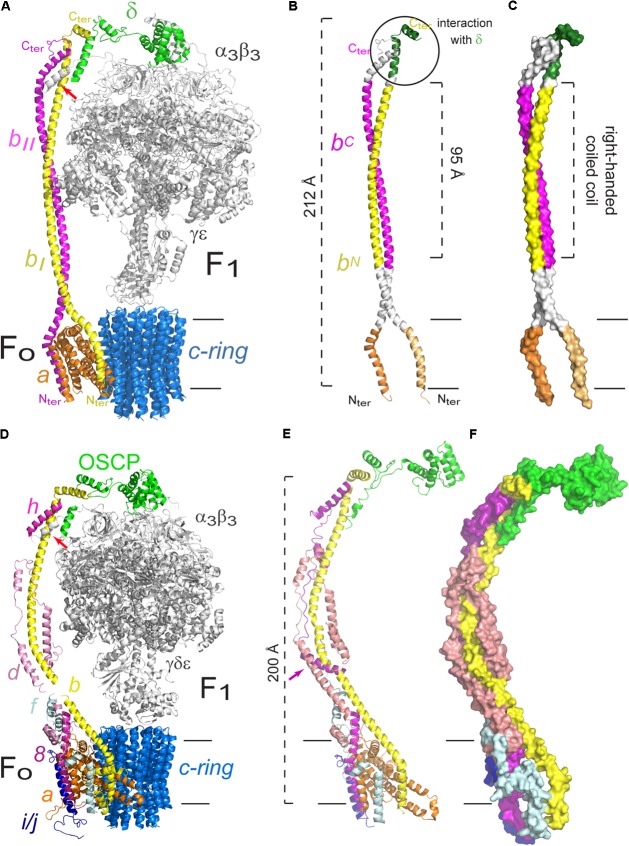
Peripheral stalk of prokaryotic and eukaryotic F-ATPases. **(A)** Three dimensional model of the enzyme of *Escherichia coli* in which the peripheral stalk and the F_O_ sector are colored. **(B)** Model that highlights the different domains of the *b*_2_ dimer. The C-terminus (C-ter) and N-terminus (N-ter) of subunit *b* are indicated. **(C)** Model to illustrate the right-handed coiled coil domain of the *b*_2_ dimer. The model used in **A–C** corresponds to the structural data deposited with the PDB 5T4O (Sobti et al., 2016). **(D)** Three dimensional model of the enzyme of *Saccharomyces cerevisiae* in which the peripheral stalk and the F_O_ sector are colored. The helix of subunit α that contacts *b*, *d* and F6 is indicated with a red arrow. **(E)** Peripheral stalk of the yeast ATPase highlighting its components. The additional helix of subunit *h* is indicated with a purple arrow. **(F)** Surface representation to illustrate the coiled coil interactions in the extrinsic part of the yeast peripheral stalk. The model used in **D–F** corresponds to the structural data deposited with the PDB 6CP6 (Srivastava et al., 2018). The black horizontal lines indicate the mitochondrial inner membrane.

The simplest known version of the F-type ATPase is the bacterial enzyme: subunits α_3_β_3_ of the catalytic core, subunits γ, 𝜀 of the central stalk, the membrane-embedded subunits *a* and a *c*-ring (*c*_x_), and a partially membrane-bound *b*_2_ dimer forming the peripheral stalk along with subunit δ (Weber, 2006; **Figure 2A**). The proton channel is formed by subunit *a* and by the interface of subunit *a* to the *c*-ring. The peripheral stalk of the enzyme from *E. coli* has been divided in four domains: (i) the N-terminal domain that crosses the membrane and interacts with subunit *a* (Dmitriev et al., 1999; Stalz et al., 2003), (ii) the tether domain, which contains residues that contact cytoplasmic loops of the *a* subunit (McLachlin et al., 2000), (iii) the dimerization domain, and (iv) the C-terminal domain, through which it interacts with subunit δ (known as subunit OSCP in eukaryotic enzymes) (McLachlin et al., 1998; Dunn et al., 2000). Subunit δ interacts with catalytic subunit α at the top of the enzyme (Rubinstein and Walker, 2002; Carbajo et al., 2007). It has been determined that the interaction OSCP-α is strong enough to resist the torque generated by the movement of the rotor (Weber et al., 2004).

Subunit *b* of *E. coli* has been studied extensively, and this has derived in a better understanding of the structure and function of this protein and, consequently, of the peripheral stalk. Small-angle X-ray scattering studies revealed that the dimerization domain is limited to residues 62–122, and that this part of the protein in solution forms an extended dimer of approximately 95 Å (Del Rizzo et al., 2002; **Figure 2B**). Mutations in the dimerization domain result in an assembled enzyme but a lack of oxidative phosphorylation (Cipriano et al., 2006). This observation suggests that the peripheral stalk of the ATPase from *E. coli* has a role beyond that of joining F_O_ with F_1_, for which the correct interaction of the dimer subunits is necessary (Del Rizzo et al., 2006). This possibility has also been explored in the F-ATPase of yeast, in which mutations in the transmembrane segment of subunit 4 (*b*) of the peripheral stalk impact the coupling of proton translocation with catalysis (Razaka-Jolly et al., 1994).

The *b*_2_ dimer associates via a right-handed coiled coil due to the presence of a conserved hendecad repeat in both *b* subunits of *E. coli* (**Figure 2C**), as well as in those from other prokaryotic organisms (Del Rizzo et al., 2002). The study of chimeras of subunit *b* has shown that it has functional tolerance as long as the residues involved in the dimerization are substituted by others that fit the hendecad repeat pattern and the resulting helices have a right-handed coiling; left-handed coiling structures assembled but were unable to support oxidative phosphorylation (Bi et al., 2008).

It has been proposed that each *b* subunit has a different role in the *E. coli* enzyme, given by the interactions each one establishes. The ATP synthase is an asymmetric enzyme due to the stoichiometry of its subunits, subunit *b* is the only one present in two copies and so the interactions of it with each monomer cannot be the same. Accordingly, it has been proven that the *b*_2_ dimer is intrinsically asymmetric and that the arrangement of its helices tends to be “offset” (Del Rizzo et al., 2006; Claggett et al., 2009). This topology has two important consequences: one of the helices of the dimer is skewed toward the N-terminus (b_N_) and the other toward the C-terminus (b_C_), so the residues occupying these positions are in different microenvironments, thus confirming that the interactions of each subunit *b* are different (Wood and Dunn, 2007).

The study of the individual interactions of each *b* subunit has been approached with crosslinking experiments (Brandt et al., 2013; Deleon-Rangel et al., 2013). It was found that the C-terminus of one of the subunits (b_I_) is the part involved in the interaction with δ (see **Figure 2B**). This b_I_ subunit (or b_N_) is the closest to subunit α and is in contact with subunit *a* in the membrane. The other *b* subunit (b_II_ or b_C_) was found in close proximity to subunit β. Taken together, these results confirm the asymmetric nature of the dimer and demonstrate that each monomer has a different role and position in the enzyme (Brandt et al., 2013). This asymmetry was further confirmed with the high resolution structures obtained for the *E. coli* enzyme by cryo-electron microscopy, which show the peripheral stalk contacts alternatively the three α subunits via their N-terminal helices but in a clearly asymmetrical fashion, using a different interface for each of them. Furthermore, the N-terminus of the *b* subunits bifurcates closely above the membrane to then separate in two helices within the membrane which contact subunit *a* from two sides (see **Figure 2A**; Sobti et al., 2016). These structures have showed, for the first time for an F-ATPase, the complete homodimeric coiled coil structure of the peripheral stalk, which spans almost the entire complex (212 of 232 Å) (Sobti et al., 2016).

#### The Peripheral Stalk of Yeast and Mammals Mitochondrial F-ATPase

The peripheral stalk of the F-ATPase of mammals and yeast shares the same subunit composition, with the exception of subunit *h* of yeast, which only has a 20% similarity with its bovine equivalent, F6 (Velours et al., 2001; Fujikawa et al., 2015), but the latter is sufficient to substitute the absence of subunit *h*, as shown by complementation experiments in *S. cerevisiae* (Velours et al., 2001). As is the case in the bacterial enzyme, the C-terminal end of the eukaryotic subunit *b* interacts with the C-terminal end of OSCP (equivalent to bacterial subunit δ) (**Figure 2D**; Rubinstein and Walker, 2002; Rees et al., 2009; Hahn et al., 2016). In the bovine enzyme, the exposed part of subunit *b* maintains interactions with subunits *d* and F6, all mediated by coiled coils, which result in an extensive and stable interaction between subunits OSCP-*b*-F6 that spans the complete length of the peripheral stalk, as shown by the crystallographic structure of the soluble section of the enzyme (Rees et al., 2009). The structure of the *S. cerevisiae* enzyme (at 3.6 Å) shows that subunit *h* has an additional helix, not present in its mammalian equivalent, that is involved in interactions with subunits *b* and *d* (Srivastava et al., 2018; **Figure 2E**).

A high resolution structure of the dimeric enzyme of the yeast *Yarrowia lipolytica* was obtained from X-ray diffraction data (3.5 Å) and cryo-electron microscopy images (6.2 Å) (Hahn et al., 2016). The sections obtained with the best resolution by cryo-electron microscopy were both the exposed and the transmembrane parts of the peripheral stalk. This model showed contacts that had not been described previously, such as the interaction of the N-terminal end of subunit α with subunits *b*, *h* and the N-terminal end of OSCP, all of which define the union of F_1_ with the peripheral stalk (see **Figure 2D**). The most recent structure of the *S. cerevisiae* enzyme, obtained by cryo-electron microscopy, shows that the N terminus of each α subunit interacts and securely anchors subunit OSCP to the top of F_1_. Furthermore, a helix from one of the α subunits (the one known as α_TP_) makes contacts with helices from subunits *b*, *d*, and *h* (Srivastava et al., 2018). These structures show that the peripheral stalk is securely attached to the catalytic core of the enzyme, not only indirectly through a *b*-OSCP interaction but also by direct contact of three peripheral stalk subunits with a catalytic subunit.

As for the contacts between subunits of the peripheral stalk and subunits located in the membrane section of the enzyme, crosslinking experiments with the bovine enzyme showed that the membrane subunit A6L (also called ATP8 in mammals and 8 in yeast) is in close proximity to subunits *b*, *d*, and F6 through its C-terminus, which extends 70 Å from the membrane to reach the peripheral stalk (Lee et al., 2015). The C-terminal region of subunit 8 in yeast has interactions with subunits *b* and *h* (Stephens et al., 2003). It has been proposed that subunit A6L/8 derived from one of the bacterial *b* subunits and is truncated in mammals and yeast, since there are four conserved residues (MQPL) in their N-terminal region (Hahn et al., 2016). Recently, He et al. (2018) have suggested that subunits 6.8PL and DAPIT of the human enzyme are functional orthologs of yeast subunits *i*/*j* and *k*, respectively, which would mean that the yeast and mammalian enzymes can be considered identical in composition. Subunit *f* is located in the F_O_ section in the bovine and yeast enzymes (Collinson et al., 1994; Spannagel et al., 1997), and has been found to interact with subunit *b* by crosslinking experiments (Spannagel et al., 1998). Finally, as in the bacterial enzyme, the base of the peripheral stalk of the enzyme of bovine and yeast contacts the F_O_ section by a *b*–*a* interaction (Spannagel et al., 1998), which has been confirmed with the high resolution structures obtained to date (Baker et al., 2012; Zhou et al., 2015; Hahn et al., 2016; Sobti et al., 2016; Guo et al., 2017; Srivastava et al., 2018; see **Figure 2D**). A recent high resolution structure of the yeast ATPase obtained by cryo-electron microscopy (at 3.6 Å) has shown the arrangement of the dimeric F_O_ section (Guo et al., 2017). In this structure, subunit *b* is shown to have one transmembrane helix that forms a domain with subunits *e* and *g* and that this domain is connected to its second transmembrane helix by a loop. Subunits *f* and *h* both interact with the peripheral stalk and are thus considered part of this structure: the N-terminal portion of subunit *f* contacts the exposed part of subunit *b*, and subunit 8 has a transmembrane helix in contact with one of the helices of subunit *a* and its C-terminal portion contributes to the formation of the base of the peripheral stalk. Finally, the C-terminal part of subunit *d*, which had not been resolved in previous structures, wraps around subunits 8 and *b* at the base of the peripheral stalk (Guo et al., 2017; **Figures 2E,F**). All of these inter-subunit contacts show how the peripheral stalk is anchored to the membrane section of the enzyme, and how this can be achieved through one interaction, as is the case in the bacterial enzyme, or through several, as is the case in mammals and yeast, both with the same result. Besides these subunit-subunit contacts in the membrane sector, the peripheral stalk also attaches to the F_1_ sector to fulfill its role as the stator of the enzyme.

#### The Peripheral Stalk of Protozoan Mitochondrial F-ATPase

**FIGURE 3:**
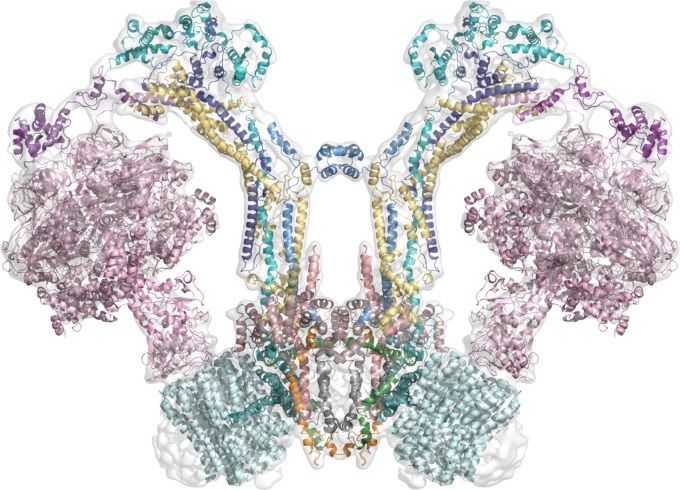
Working model of the dimeric mitochondrial ATPase of *Polytomella* sp. The image shows the working model of the 3D structure of the enzyme fitted in the EMD-2852 map contoured at 6 sigma (Allegretti et al., 2015). Color scheme: F_1_ sector in pink; OSCP in violet; Asa2 in cyan; Asa4 in deep purple; Asa7 in sky blue; Asa1 in yellow; Asa3 in brown (dirty violet); Asa5 in salmon; Asa6 in gray; Asa8 in orange; Asa9 in leaf green; subunit a in deep teal and *c*-ring in pale cyan.

In stark contrast with the F-ATPases described so far, the mitochondrial enzyme of chlorophycean algae such as *Chlamydomonas reinhardtii* and *Polytomella* sp. has several striking features, one of which is the presence of a robust peripheral stalk formed by nine subunits named Asa (ATP Synthase Associated) (Vázquez-Acevedo et al., 2006; van Lis et al., 2007; Cano-Estrada et al., 2010), some of which (Asa6 and Asa9) are involved in the dimerization of the enzyme (Villavicencio-Queijeiro et al., 2009; Cano-Estrada et al., 2010; Lapaille et al., 2010; Sánchez-Vásquez et al., 2017). This enzyme has no clear homologs for any of the subunits that typically form the peripheral stalk, however, some equivalent interactions have been found: subunit Asa1 contacts the C-terminal end of subunit OSCP, which is reminiscent of the *b*-OSCP interaction in the other F-ATPases (Colina-Tenorio et al., 2016). Some of the Asa subunits (Asa1, Asa2, Asa4, and Asa7) are predicted to adopt coiled coil structures (Miranda-Astudillo et al., 2014), which is consistent with the nature of subunits *b* in other F-ATPases and subunits E and G of the peripheral stalk of A and V-type ATPases (see sections “The Peripheral Stalk of A-ATPases” and “The Peripheral Stalk of V-ATPases”). Recently, an Asa6-*a* interaction was shown in a three dimensional map generated with cryo-electron microscopy, and it was found that subunit Asa6 has a V-shape similar to that of the N-terminal part (transmembrane) of subunit *b* (Klusch et al., 2017) and that the Asa6 subunit together with the H5/H6 hairpin of subunit *a* form the luminal half-channel. Although a structural map obtained by cryo-electron microscopy for the *Polytomella* enzyme is available at 7 Å resolution (Allegretti et al., 2015) there is currently no high resolution data to distinguish structural details of the Asa subunits in the peripheral stalk, nevertheless, numerous biochemical studies have established several near-neighbor relationships between the Asa subunits and other constituents of the peripheral stalk. Thus, based on the low resolution map available and the biochemical evidence, a model depicting a possible location of the different subunits can be inferred (**Figure 3**).

**FIGURE 4:**
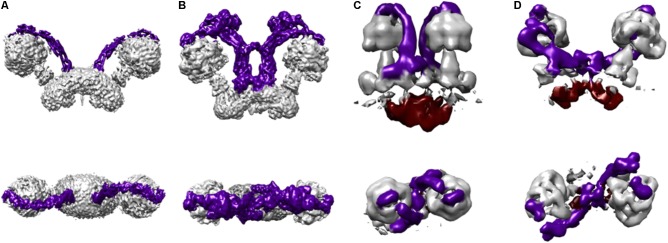
Protozoan and metazoan type dimers. Three dimensional maps of dimeric ATP synthases from **(A)** *Saccharomyces cerevisiae* (EMD 7067) (Guo et al., 2017) representing a metazoan-type dimer, **(B)** *Polytomella* sp. (EMD 2852) (Allegretti et al., 2015), **(C)** *Paramecium tetraurelia* (EMD 3441) (Mühleip et al., 2016), and **(D)** *Euglena gracilis* (EMD 3559) (Mühleip et al., 2017) representing the protozoan-type dimers. The colors in the schematizations represent: peripheral stalks in dark purple and the inter membrane space density below the *c*-ring in deep red **(C,D)**.

Recently, a convenient new separation of ATP synthase complexes was put forward by Mühleip et al. (2017) that distinguishes the metazoan-type dimers from the protozoan dimers or those from unicellular algae, the latter included in the protozoan-type. This separation came about when some striking differences became evident with the generation of three-dimensional structures, although the biochemical and genetic evidence, as well as low resolution negative stain projection images, much preceded these structures. Metazoan and protozoan-type dimers differ by both the structure of their peripheral stalks and by their dimeric interface (Mühleip et al., 2017). Metazoan-type enzymes have a V-shape and include mammalian and fungi ATPases. Some examples of protozoan-type dimers include the F-ATPases from: *Polytomella* sp. (Cano-Estrada et al., 2010; Dudkina et al., 2010), *Trypanosoma brucei* (Zíková et al., 2009), *Tetrahymena thermophila* (Balabaskaran Nina et al., 2010), *Paramecium tetraurelia* (Mühleip et al., 2016), and *Euglena gracilis* (Yadav et al., 2017), all of which have atypical features (**Figure 4**). As described above, the general architecture of the mammalian, yeast and bacterial enzymes (metazoan-type) is essentially the same and all of their subunits share homology. It should be noted that the bacterial enzyme has only been detected in monomeric form and none of the subunits involved in the dimerization in other organisms have been identified, but since it shares general features with metazoan-type enzymes and its subunits share homology with fungi and mammalian subunits, it has been included in the metazoan-type group. In contrast with this, the peripheral stalk structures of protozoan-type dimers are highly divergent and so far no homologs for their subunits have been identified in the databases.

#### The Peripheral Stalk of Chloroplast F-ATPase

The F-ATPases of chloroplasts have a very similar subunit composition to the bacterial enzyme (Seelert and Dencher, 2011) and their peripheral stalk is formed by a *bb*’ dimer (also called subunits I and II), both of these subunits are structurally and functionally similar to the bacterial dimer (Rühle and Leister, 2015). However, in both chloroplasts and cyanobacteria, these subunits are not identical and each one has different secondary structure and dimerization domain (Poetsch et al., 2007). The peripheral stalk of this type of ATPase was first detected through the averaging and analysis of electron microscope images (Böttcher et al., 1998). A three dimensional map was obtained later on at 20 Å resolution (Mellwig and Böttcher, 2003). This reconstruction was generated based on cryo-electron microscopy and the peripheral stalk was found to be a thin structure with more prominence in the parts that contact F_1_ and F_O_. Mellwig and Böttcher suggest there must be communication between F_1_ and F_O_, and propose the peripheral stalk as the structure responsible for that communication.

A three-dimensional structure for a chloroplast F-ATPase was recently obtained by cryo-electron microscopy at a resolution of 2.9–3.4 Å (Hahn et al., 2018). The observed peripheral stalk structure is highly similar to the bacterial one: subunits *b* and *b*’ have a helical conformation and associate through a right-handed coiled coil that ends right above the membrane surface, where the helices separate and cross the membrane while clamping the *a* subunit to the *c*-ring, just as it happens in the *E. coli* ATPase. Also reminiscent of the bacterial enzyme, the C-terminus of subunit *b* interacts with the C-terminus of subunit δ, which is shown to be formed by a four-stranded mixed β sheet and two α helices, all of which provide the surface for subunit *b* to attach. Finally, as was also observed in the yeast structures, the N-terminus of one of the α subunits interacts with subunit *b’* (Hahn et al., 2018). Once again, all of these interactions confirm the conserved nature of the overall architecture of the peripheral stalk as well as its role as the stator of the complex given its contacts with both sections F_1_ and F_O_.

### The Peripheral Stalk of A-ATPases

Archaea have adapted to the most extreme living conditions in terms of temperature, salinity, pressure, pH, etc. Many of them live in substrates that do not allow the synthesis of 1 mole of ATP per mole of substrate (Mayer and Müller, 2014), which is why their energy conservation strategies are different to those of bacteria and eukaryotes, and involve a chemiosmotic mechanism in which their metabolism is coupled to the generation of sodium or proton gradients to drive the synthesis of ATP (Deppenmeier and Müller, 2007). Despite these differences, ATP synthesis occurs quite, similarly, to how it occurs in F-ATPases, and the overall architecture of the enzyme among the studied species is conserved (Mayer and Müller, 2014).

**FIGURE 5:**
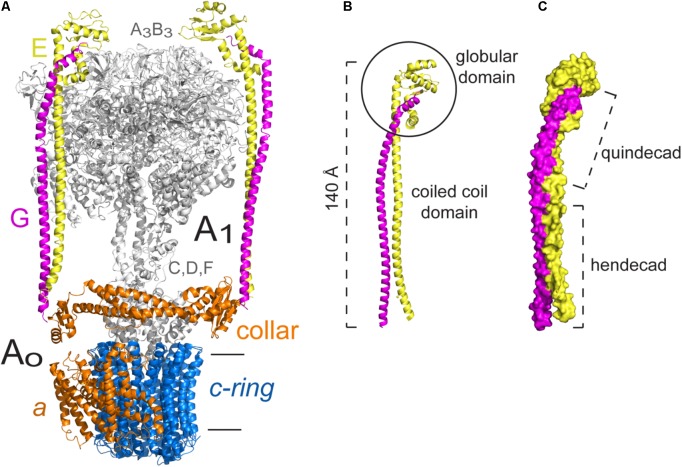
Peripheral stalk of the *Thermus thermophilus* A-ATPase. **(A)** Three dimensional model of the archaeal enzyme (Schep et al., 2016) PDB 5GAR, in which the peripheral stalks, the collar-like structure made by subunit *a* and the *c*-ring are colored. **(B)** Model of the EG heterodimer in which the coiled coil domain and the globular domain are indicated. **(C)** Model of the EG heterodimer that illustrates the right handed coiling of the helices and the two types of coiling that result from the hendecad motifs in both subunits, and quindecad repeat motifs in subunit G. The model used in **B,C** corresponds to the structural data deposited with the PDB 3V6I (Stewart et al., 2012). The black horizontal lines indicate the mitochondrial inner membrane.

A-ATPases are formed by the sectors A_1_ and A_O_, in this case joined by two peripheral stalks (**Figure 5A**). Sector A_1_ contains the catalytic domain A_3_B_3_ and subunits C, D, and F of the rotor; and the membrane sector A_O_ forms the channel for the translocation of ions and protons with subunits *a* and *c*. Subunit D extends through the hexamer formed by subunits A and B, thus connecting the site of catalysis with the site of proton translocation through subunits *a*–*c* in sector A_O_ (Grüber et al., 2014). Subunit A has additional alpha helices in its C-terminus and a “non-homologous region” in its N-terminus region, both of these characteristics are shared with its equivalents in V-ATPases (Radermacher et al., 2001), but not with F-ATPases. An outstanding feature of A-ATPases is the size variation of the *c*-ring and its capacity to couple the binding of different ions with ATP synthesis (Grüber et al., 2014).

Both peripheral stalks of A-ATPases are formed by heterodimers of subunits E and G. In solution, these subunits adopt a helical structure, just as the proteins that form the peripheral stalk in other ATPases (Kish-Trier et al., 2008). The EG heterodimer has a coiled coil structure along its N-terminal region and a globular structure on its C-terminus, the latter has been shown to interact with the N-terminal end of subunit B of the catalytic core by magnetic resonance studies (Kish-Trier and Wilkens, 2009). The interaction of a component of the peripheral stalk with one of the catalytic subunits appears to be a conserved feature among rotary ATPases, since it has been described for every type of enzyme in the family. Subunit G shares some similarity with the extramembranal part of subunit *b* of F-ATPases, which suggests a common origin (Hunt and Bowman, 1997). Furthermore, the crystallographic structures of subcomplexes corresponding to the peripheral stalk of an A-type ATPase (Lee et al., 2010) and an F-type ATPase (Dickson et al., 2006) show that subunits G and *b* have a very similar elongated helical structure (Muench et al., 2011). Sobti et al. (2016) found that, although sequence identity is low (22%), the general fold of the soluble portion of the *E. coli* peripheral stalk is very similar to that of the *T. thermophilus* A-ATPase (Lee et al., 2010), which indicates a strong evolutionary pressure for proteins to adopt this type of fold (Sobti et al., 2016).

The first three dimensional structures of complete A-ATPases were obtained with reconstructions from electron microscopy images, at 23 Å for the H^+^-ATPase of *Thermus thermophilus* (Bernal and Stock, 2004) and at 18 Å resolution for the A-ATPase of *Methanococcus jannaschii* (Coskun et al., 2004). With these structures the presence of two peripheral stalks was established, as was their connection with both A_1_ and A_O_. Additionally, it was found that these peripheral stalks are asymmetric, one is bent toward A_1_ and the other has a more vertical disposition. This observation was later confirmed when the crystallographic structure of subunit E was obtained at 3.6 Å for *Pyrococcus horikoshii* (Balakrishna et al., 2012). In this work, when adjusting the obtained structure of subunit E into the three dimensional map of the enzyme, a better fit was found for the bent peripheral stalk, while the same subunit crystallized previously (Lee et al., 2010), showed a better fit to the vertical stalk (Balakrishna et al., 2012).

The crystal structure of the EG heterodimer of the H^+^-ATPase of *T. Thermophilus* obtained at a 3.1 Å resolution clearly showed the structure and topology of these subunits (Lee et al., 2010). Both subunits have an enriched repetitive sequence of alanine, leucine, glutamate and arginine residues, and they assemble into an elongated heterodimer with two distinguishable domains: a 140 Å-long right-handed coiled coil region and a globular region formed mainly by the C-terminus of subunit E (**Figure 5B**). The coiled coil region is formed due to a hendecad repeat pattern in the N-terminus of both subunits, however, in subunit G, this pattern changes to a quindecad repeat that results in a tighter coiling (**Figure 5C**). The structure of heterodimer EG was fitted into the three dimensional map of the complete enzyme (Bernal and Stock, 2004), which revealed that it is specifically the N-terminus of subunit E the part in contact with the catalytic core (Lee et al., 2010).

Contrary to what happens in F-ATPases, the peripheral stalks of A-ATPases do not cross the membrane but are anchored to a collar-like structure in the extramembrane base of the complex, and extend from there to the A_3_B_3_ hexamer (Bernal and Stock, 2004; Grüber et al., 2014; see **Figure 5A**). The collar structure is formed by the N-terminal region of subunit *a* (this subunit has also been called *I* in these enzymes), which has an exposed globular domain that can interact with both peripheral stalks (Vonck et al., 2009; Lau and Rubinstein, 2012). The EG heterodimer can be considered to be functionally similar to the *b*_2_ homodimer of the bacterial F-ATPase, since it is also an asymmetric dimer and each subunit has a different role: subunit E mediates the interaction with the catalytic subunits and subunit G stabilizes the peripheral stalk (Lee et al., 2010; Grüber et al., 2014).

### The Peripheral Stalk of V-ATPases

V-type ATPases couple the hydrolysis of ATP with ion transport and they are involved in many cellular processes: vesicular traffic, processing and degradation of proteins, coupled transport of small molecules and acidification of organelles, among others (Stransky et al., 2016). The V_1_ sector includes the catalytic core A_3_B_3_ where ATP is hydrolyzed, and the central rotor formed by subunits D and F. The V_O_ sector includes the *c*-ring oligomer, subunit *d* and the membrane part of subunit *a* (Forgac, 2007). Both sections are joined by three peripheral stalks formed by heterodimers of subunits E and G, which are anchored to the base of the complex through a collar-like structure made by subunits C, H and the soluble domain of subunit *a* (Rawson et al., 2016).

A particular feature of V-ATPases is their regulatory mechanism, which involves the peripheral stalks. *In vivo* experiments of V-ATPase from insects (Sumner et al., 1995) and yeast (Kane, 1995) suggested that V-ATPases are able to disassemble and reassemble in response to extracellular stimuli. Both *in vivo* and *in vitro* experiments suggest that the regulation happens as a result of a rearrangement of the subunits of the enzyme (Oot and Wilkens, 2012; Tabke et al., 2014). The exact mechanism is still unknown, but the evidence suggests that the subunits of the peripheral stalk should allow some degree of movement to the complex, either to disassemble or reassemble, or to accommodate the rearrangement of its subunits (Oot et al., 2017). Studies of the structure of the EG dimer and an EGC subcomplex have shown that the interaction between these subunits is stronger when they are part of the holoenzyme than when they are in solution, which indicates that a conformational change of EG/EGC can occur at some point of the regulation (Diepholz et al., 2008).

**FIGURE 6:**
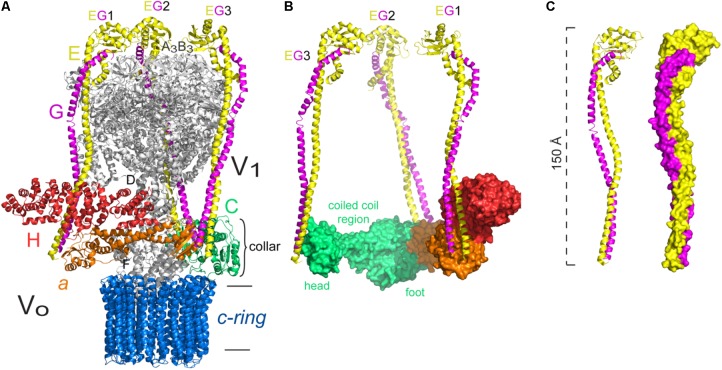
Peripheral stalk of the *Saccharomyces cerevisiae* V-ATPase. **(A)** Three dimensional model of the yeast enzyme in which the peripheral stalks and the subunits of the collar-like structure are colored. **(B)** Model that illustrates the interaction of each peripheral stalk (EG1, EG2, EG3) with the subunits of the collar. The different sections of subunit C are indicated. **(C)** Models of the EG heterodimer to illustrate the right handed coiling of the helices. The model used in **A–C** corresponds to structural data deposited with the PDB 3J9V (Zhao et al., 2015). The black horizontal lines indicate the mitochondrial inner membrane.

As mentioned above, sectors V_1_ and V_O_ are joined by three peripheral stalks (**Figure 6A**). These stalks were first observed in electron microscopy images (Boekema et al., 1997; Ubbink-kok et al., 2000; Wilkens et al., 2005; Muench et al., 2009) and a detailed model of the subunits and their interactions was obtained with the crystallographic structure of the EGC subcomplex of the yeast V-ATPase (Oot et al., 2012), which crystallized in two different conformations at 2.91 and 2.82 Å. These structures clearly show that two of the peripheral stalks (EG1 and EG2) join the highest part of the enzyme with the exposed N-terminus of subunit *a*, and the third stalk (EG3) interacts with subunit C, which has no homologs in A and F-type ATPases (**Figure 6B**). The crystallographic structure of subunit C showed that it is formed by two globular domains, which have been called “head” and “foot,” separated by a coiled coil stretch (Drory et al., 2004; **Figure 6B**). It was later determined that the EG-C interaction is crucial to maintain the stability of the EG heterodimer, and that the interaction is mediated by the “head” domain of subunit C (Oot and Wilkens, 2010). The complete structure of the V-ATPase of *Saccharomyces cerevisiae* was obtained at 11 Å resolution from cryo-electron microscopy studies of protein particles in ice (Benlekbir et al., 2012). This structure shows the contact of the three peripheral stalks with V_1_, given by the N-terminal end of the E subunits with the B subunits of the catalytic subunits (see **Figure 6A**). It can also be seen that each EG heterodimer interacts with different subunits of the collar-like structure (subunits *a*, C, and H): EG1 interacts with the N-terminal ends of subunits *a* and H; EG2 with the N-terminal end of subunit *a* and the “foot” of subunit C; and EG3 only contacts the “head” of subunit C (see **Figure 6B**). In this enzyme the only contact between a peripheral stalk and the membrane sector is given by the interaction of EG2 with subunit *a* (Benlekbir et al., 2012), since the rest of the subunits that form the collar are not membrane subunits. This can be contrasted with A-ATPases in which the collar is formed exclusively by subunit *a* contacting both peripheral stalks (see **Figure 5**).

The EG heterodimer of yeast V-ATPase forms a long structure (of approximately 150 Å) and when bound to subunit C, the subcomplex EGC_head_ (of approximately 170 Å) maintains an elongated shape (Oot et al., 2012; **Figure 6C**). The interaction between subunits E and G is stronger in the N and C-terminal ends and weaker in the middle of the helices, and it is due to hendecad repeat patterns that cause a right-handed coiled coil interaction (Oot et al., 2012). The presence of this characteristic structure reinforces the idea that the right-handed coiling is a conserved feature of the proteins that build the peripheral stalks of rotary ATPases (Stewart et al., 2013).

## The Roles of the Peripheral Stalk

All the interactions described so far clearly establish the role of the peripheral stalk as the structure responsible for connecting the two sectors that form ATPases, the membrane sector and the catalytic core. Having discussed that, the following section describes the different roles that have been attributed to the peripheral stalks and the latest proposals based on the growing wealth of structural information.

### The Role of the Peripheral Stalk in the Flexibility of the Complex

Evidently, rotary ATPases are dynamic structures that exhibit some degree of flexibility that allows all the movements that are necessary for the enzyme to function correctly (Walker and Dickson, 2006; Neukirch et al., 2008; Stewart et al., 2012, 2013). The notion that the peripheral stalk is a flexible structure has been controversial and has evolved. The flexibility property was first assigned to the *b* subunits of the F-ATPase of *E. coli*, based on experiments in which residues were added or removed from these subunits and the enzyme remained functional (Sorgen et al., 1998, 1999). Years later it was proposed that the peripheral stalk is a rigid structure (Dickson et al., 2006; Rees et al., 2009), and that the need for flexible elements is fulfilled by other components of the enzyme (Wächter et al., 2011). In recent years, cryo-electron microscopy studies suggest that the peripheral stalk, to a certain extent depending on the type of ATPase, is indeed a flexible structure (Mazhab-Jafari and Rubinstein, 2016).

Subunits *a* and *c* of the F_O_ sector form two aqueous half channels that define the path followed by protons, which drive the movement of the rotor (*c*-ring + γδ𝜀) in order for catalysis to occur (F_1_). This fact, as well as the key residues involved in proton translocation, were first proposed by Vik and Antonio (1994) based on mutagenesis experiments, and both were confirmed over 20 years later by cryo-electron microscopy studies (Allegretti et al., 2015; Guo et al., 2017). It is well known that each complete turn of the rotor generates, on average, three ATP molecules (Yasuda et al., 1998), and that each turn requires the translocation of a certain number of protons, depending on the number of *c* subunits present in the *c*-ring (Pogoryelov et al., 2012). This difference or asymmetry between what goes into the complex and what comes out (8–15 H^+^:3 ATP) requires the temporal storage of energy during the movement and its gradual release to drive each 120° turn of the rotor (Cherepanov et al., 1999; Walker and Dickson, 2006; Junge et al., 2009). Another way of looking at this phenomenon is to consider rotation steps: sector F_1_ has a three-step rotation (given by the three β subunits), while sector F_O_ has an 8–15-step rotation (depending on the number of *c* subunits). This difference has been called rotational asymmetry and it is buffered by the transmission of elastic energy between the two sectors of the enzyme (Saroussi et al., 2012). Taken together, these observations imply that there have to be flexible elements in the enzyme capable of storing and transmitting elastic energy.

Experiments performed with single molecules of the F-ATPase of *E. coli*, in which certain domains are “stiffened” by artificial disulfide bonds and their elasticity is measured, identified the lower part of the rotor (γ𝜀 + *c*-ring) as an elastic domain (Sielaff et al., 2008). Other studies have evaluated the magnitude and determinants of the elasticity of the peripheral stalk of the bacterial ATPase, comparing wild type and mutant enzymes with modified *b* subunits, and have concluded that the peripheral stalk is a rigid structure and the most elastic elements are located in the central rotor and the lever of subunit β (Wächter et al., 2011). These authors suggest that, in the *E. coli* enzyme, the peripheral stalk works as a scaffold between F_O_ and F_1_, and that the rotor (at least ten times more flexible) is responsible for the transmission of elastic energy between them.

**FIGURE 7:**
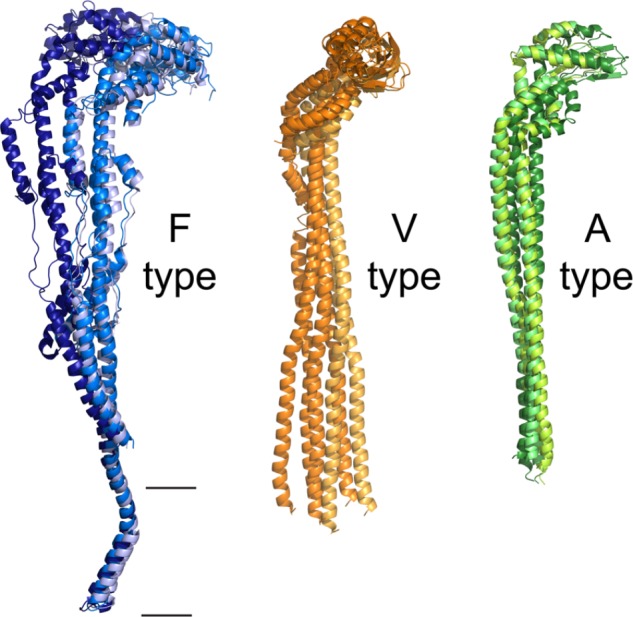
Flexibility of the peripheral stalk of rotary ATPases. Flexibility of the peripheral stalk illustrated with the transitions it goes through during the process of rotational catalysis. **(A)** Models that correspond to the transitions of an F-ATPase peripheral stalk; PDBs 5ARI, 5ARA, 5FIL from Zhou et al. (2015). The membrane section in these models is not accurately represented due to the resolution of the maps. Higher resolution maps have been obtained but only in one rotational state and thus are not useful to illustrate the flexibility of the peripheral stalk. **(B)** Models that correspond to three rotational states of one of the peripheral stalks of the V-ATPase of *S. cerevisiae*; PDBs 3J9T, 3J9U, 3J9V from Zhao et al. (2015). **(C)** Models that correspond to three rotational states of one of the peripheral stalks of the A-ATPase of *T. thermophilus*; PDBs 5Y5X, 5Y5Z, 5Y60 from Nakanishi et al. (2018). The black horizontal lines indicate the mitochondrial inner membrane.

In light of the latest evidence, obtained by cryo-electron microscopy, the stiffness that had been assigned to the peripheral stalk is now being reconsidered. Structures in more than one conformational state have been generated for the bovine F-ATPase (Zhou et al., 2015), which show the transitions of the enzyme. Two transitions of the peripheral stalk are visible: a bend toward the top part of the enzyme close to subunit OSCP and a bend toward the transmembrane part of subunit *b* (**Figure 7A**). The authors conclude that the flexibility and movement capacity of all the components of the enzyme (the *c*-ring showed considerable rotational flexibility) contribute to facilitate the coupling of the rotor movement (F_O_) with catalysis (F_1_). Similarly, cryo-electron microscopy studies from the V-ATPase of *S. cerevisiae* allowed the reconstruction of 3D structures in three conformational states (Zhao et al., 2015). In this enzyme, as in the bovine enzyme, most of the subunits show conformational changes. The obtained structures of the yeast enzyme show that the helical part of rotor subunit D (equivalent to subunit γ of F-ATPases) remains rigid during rotation, but the part in contact with subunit *d* can bend. It is also evident that the catalytic subunits A and B press on subunits E and G of the peripheral stalks, which then bend along their coiled coil regions (**Figure 7B**). Even more flexibility is observed when considering the EG-C interaction, since subunit C can twist without losing contact with the peripheral stalks.

In contrast with what was observed in F and V-type ATPases, the analysis of the rotational states of the A-ATPase of *T. thermophilus* (Schep et al., 2016) revealed that the conformational changes of its subunits are minimal, which would suggest a less flexible enzyme. The authors argue that this can be due to the fact that this enzyme has a larger rotational asymmetry (3:12) compared to the one of the yeast V-ATPase (3:10) and bovine F-ATPase (3:8), which may cause it to adopt an energetically favorable rotational state in which most of the images are obtained, resulting in an apparent lack of flexibility. Recently, a larger data set of single particle images obtained by Nakanishi et al. (2018) allowed the identification of the missing third rotational state of the enzyme of *T. thermophilus*; taken together, the structures show there is a dynamic rearrangement of the peripheral stalks in the transitions between each rotational state (Nakanishi et al., 2018; **Figure 7C**).

### The Role of the Peripheral Stalk in the Stability and Assembly of the Complex

The study of the role of the peripheral stalk in the assembly process of the complex and how it contributes to its stability refers mostly to F-ATPases. The study of different mutations in the F-ATPase of *E. coli* revealed that subunit δ (equivalent to mitochondrial subunit OSCP) is essential for the assembly of the *b*_2_ dimer with the rest of the complex, independent of its interaction with subunit α (Hilbers et al., 2013). Additionally, the authors conclude that subunit δ is also important to join the peripheral stalk with the rotor, therefore contributing to the stability and functionality of the complex.

Most of the information available concerning the assembly of F-ATPases has derived from the study of yeast mutants. In this organism, radioactive and pulse-chase labeling experiments have allowed the elucidation of a part of the assembly process, which involves two separate sub complexes: *a*-8-peripheral stalk and F_1_-*c*_10_, which are generated in an independent but coordinated way (Rak et al., 2011). A dimeric complex named INA (Inner Membrane Assembly) was identified and proposed to act as a sort of chaperone for the assembly of the enzyme (Lytovchenko et al., 2014). The loss of this complex, composed by subunits Ina17 and Ina22, causes the dissociation of sectors F_1_ and F_O_, and it was found that subunit Ina22 associates transiently with both F_1_ and the peripheral stalk, but not with the assembled enzyme, which confirms its role as an auxiliary factor. Lytovchenko et al. (2014) propose an alternative to the assembly route proposed by Rak et al. (2011) that includes an F_1_-peripheral stalk sub complex. It was then proposed that INAC prevents premature interaction of assembly intermediates and promotes the correct assembly of the *c*-ring with subunit *a* to form the proton translocation portion of the enzyme (Naumenko et al., 2017). A recent study with null mutants of human ATPase subunits showed that, although the human and yeast ATPases are highly similar, the assembly pathways of the proton translocation channel are different (He et al., 2018; Song et al., 2018).

**FIGURE 8:**
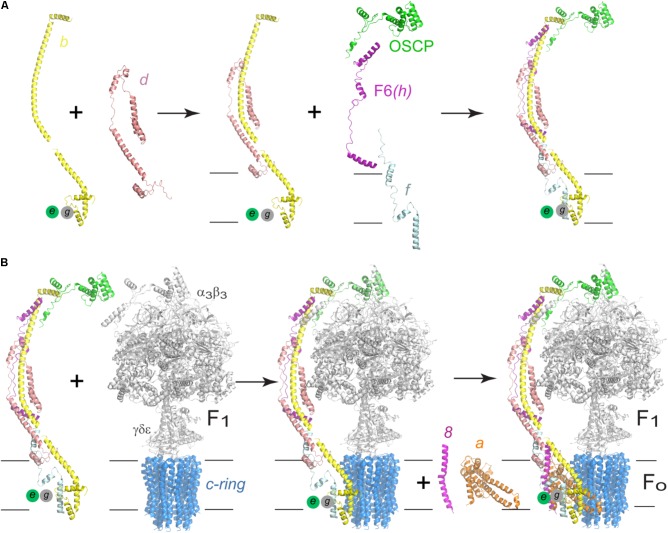
Assembly process of the human F-ATPase. **(A)** A possible assembly pathway of the peripheral stalk based on the data of Fujikawa et al. (2015) and He et al. (2018). **(B)** One of the possible assembly pathways of the complete enzyme. According to the branched model proposed by He et al. (2018) and Song et al. (2018), another possibility involves an initial *b*-*d*-F6-OSCP subcomplex that first joins F_1_-*c*-ring after which subunits *f*, *e*, and *g* recruited. The model used in **A,B** corresponds to the yeast enzyme, PDB 6CP6 from He et al. (2018). The black horizontal lines indicate the mitochondrial inner membrane.

Native electrophoresis studies have shown that human F-ATPase can assemble if subunits *a* and A6L are missing and even form oligomers, albeit unstable and in low quantities (Wittig et al., 2010). It has also been shown that human cells fail to assemble ATPase if the expression of subunit *d* is inhibited, causing the accumulation of two subcomplexes: F_1_-*c*-ring and *b*-*e*-*g*, which suggests the complete peripheral stalk is necessary to maintain the stability of the enzyme (**Figure 8A**; Fujikawa et al., 2015). Further studies of the human enzyme have provided new information regarding its assembly and a branched pathway has been proposed: one branch starts with an F_1_-*c*-ring subcomplex that is joined first by the peripheral stalk (*b*-*d*-F6-OSCP) and then by subunits *e*, *g*, and *f*. Another branch involves a complete *b*-*e*-*g*-*d*-*f* -F6-OSCP subcomplex joining F_1_-*c*-ring (**Figure 8B**). The final complex in either branch (peripheral stalk-F_1_-*c*-ring) is necessary for the addition of subunits *a* and A6L (8 in yeast) (see **Figure 8**). The complete assembled complex includes subunits DAPIT and 6.8PL (He et al., 2018; Song et al., 2018). The assembly of the peripheral stalk before it joins the F_1_ domain remains unclear, however, the evidence so far indicates that one of the earlier steps is the formation of an assembly intermediate formed by subunits *b*-*e*-*g* (Fujikawa et al., 2015; He et al., 2018).

### The Role of the Peripheral Stalk in the Dimerization and Oligomerization of F-ATPases and on Membrane Curvatures

Another one of the processes in which the peripheral arm is involved is the dimerization of the enzyme. It is currently well known that F-ATPases form dimers that are arranged in rows along the inner mitochondrial membrane (Strauss et al., 2008; Thomas et al., 2008; Davies et al., 2011), and that such oligomerization is directly involved in the morphogenesis of the mitochondrial cristae (Paumard, 2002; Dudkina et al., 2005; Fronzes et al., 2006; Davies et al., 2012; Mühleip et al., 2016). Membrane subunits *e* and *g* have been identified as responsible for the stabilization of yeast (Arnold et al., 1998, 1999; Davies et al., 2012) and bovine (Minauro-Sanmiguel et al., 2005) ATP synthase dimers. However, it has also been proposed that subunit *i* of the peripheral stalk participates in this process, since there is evidence that this subunit forms homodimers that are located close to the dimerization interface (Paumard et al., 2002), and that the enzyme can be found as a dimer in the absence of subunits *e* and *g* (Fronzes et al., 2006). In addition to this, a study of interactions monitored by FRET showed that the yeast F-ATPase is capable of forming oligomeric associations *in vivo* in the absence of subunit *e* (Gavin et al., 2005), so the authors propose the existence of two dimerization interfaces, one mediated by subunits *e* and *g*, and another mediated by the transmembrane segment of subunit *b*.

In addition to the biochemical studies, structural studies of the yeast F-ATPase have provided information about how the peripheral stalk can participate in the dimerization process. A study involving the reconstruction of the yeast enzyme (from electron cryo-tomography images at an estimated resolution of 3.7 nm) and the analysis of dimer and oligomer formation *in situ* with molecular dynamics (Davies et al., 2012; Mühleip et al., 2016) showed that ATPase monomers associate through the membrane part of the peripheral stalk and that subunits *e*, *g*, and 4 (equivalent to subunit *b*) are part of the dimerization interface and essential for that process to occur. Furthermore, the N-terminal end of subunit *g* is exposed to the mitochondrial matrix (Belogrudov et al., 1996), and crosslinking experiments have proved that this segment is in close proximity to subunit 4 (Soubannier et al., 2002). The proximity of subunits *g* and 4 is in agreement with the structure obtained by Guo et al. (2017).

**FIGURE 9:**
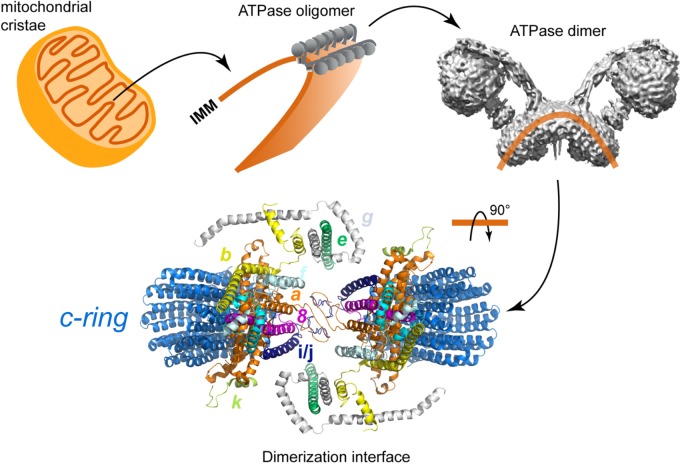
Schematic representation of the dimerization of the yeast F-ATPase. Model of the F_O_ section in which all the subunits involved in the dimerization are colored: subunit *a* in hot pink, subunit *b* in cyan, subunit *e* in brown, subunit *g* in pale blue, subunit *j* in dark blue, and subunit *k* in lemon. The dimerization interface includes the subunits proposed by Habersetzer et al. (2013) and Guo et al. (2017). The model corresponds to the structural data deposited with the PDB 6B2Z (Guo et al., 2017).

The role of subunit 4 in the dimerization/oligomerization of the yeast enzyme has been studied with directed mutagenesis of the loop that joins the transmembrane segments of this subunit (Weimann et al., 2008). This loop is necessary to organize and stabilize the neighboring subunits *a*, *e*, and *g*, and hence essential to maintain the supramolecular species of ATPase. In support of this result, and highlighting the role of the peripheral stalk in the dimerization process, there is evidence that suggests that the loss of the first transmembrane segment of subunit 4 results in a functional enzyme that is incapable of forming dimers or oligomers (Soubannier et al., 2002). Concerning the formation and maintenance of ATPase dimers, the dimerization interface of the yeast enzyme has been proposed to be stabilized by 4–4, *e*–*g*, and *a*–*a* interactions (Habersetzer et al., 2013) or by *a*/6–*i* interactions (Guo et al., 2017), but see also the work of Anselmi et al. (2018), in which dimer stabilization through 4–4 interactions has been put in doubt (**Figure 9**). The *a*–*a* interface was first demonstrated by Velours et al. (2011) with crosslinking experiments and it was recently confirmed with the high resolution structure of the dimeric enzyme, in which interactions between the C-terminal sections of two *i*/*j* subunits reinforce the *a*–*a* mediated interface (Guo et al., 2017).

Peripheral stalks could also play a role in the formation of dimer rows and cristae shaping. Two mechanisms of row formation have been described so far (Mühleip et al., 2016, 2017). The first one is found with V-shaped dimers like the ones of yeast or metazoan organisms. These V-shaped dimers display angles between monomers ranging from 56 to 120° (Minauro-Sanmiguel et al., 2005; Dudkina et al., 2006; Davies et al., 2011, 2012). It was proposed that the V-shape of the bovine ATP-synthase dimer could be induced by a “bending domain” comprising subunits *e* and *g* (Baker et al., 2012). This was later confirmed with the high resolution structure of yeast dimers (Guo et al., 2017). This cryo-EM structure also revealed that in addition to subunits *e* and *g*, the 50 residues from the N-terminus of subunit 4 also belong to this “bending domain.” As shown by molecular dynamics simulations, V-shaped dimers would impose, at the dimer interface, a local curvature on the lipid bilayer that would be favorable for the self-association of other dimers (Anselmi et al., 2018). This mode of association would be responsible for the formation of dimer rows at the ridges of lamellar cristae.

A second mechanism has been proposed for the U-shaped dimer of *Paramecium tetraurelia*, in which the two monomers are parallel and there is no membrane curvature at the dimer interface. The 2.6 nm resolution cryo-EM structure of the *Paramecium tetraurelia* dimer has clearly identified a massive intracrista domain that creates a rigid region at the base of the dimer. This intracrista domain connects the *c*-ring of one monomer to the peripheral stalk of the other monomer. Due to these interactions, the axis connecting the F_1_ sectors is tilted by 30° compared to the axis of the intracrista base (Mühleip et al., 2016). Besides, on the matricial side of U-shaped dimers, the lateral position of peripheral stalks contrasts with the more centered position of peripheral stalks of V-shaped dimers. The offset position of F_1_ sectors and the lateral position of peripheral stalks create a wider matricial region than the intracrista domain, generating a “wedge-shaped” dimer. When they associate, two consecutive wedge-shaped dimers are rotated by 8° in the direction of the row, generating a helical curvature of the membrane that matches the curvature of tubular cristae observed in *Paramecium tetraurelia* mitochondria.

Although the majority of the studies regarding the dimerization/oligomerization of ATP synthase focus on the model organisms (i.e., *S. cereviseae* and *B. taurus*), it is noteworthy that in chlorophycean algae the structural unit of the enzyme is a dimer that can form highly stable supramolecular associations (tetramers, hexamers) (Miranda-Astudillo et al., 2018). Furthermore, the peripheral stalk of the algal ATPase has an additional dimerization domain outside the membrane region (see **Figure 3**; Allegretti et al., 2015). These unique features reinforce the idea that the protozoan-type enzymes have additional characteristics, compared with the metazoan enzymes, such as dimer formation, stability, and oligomerization (Villavicencio-Queijeiro et al., 2009; Yadav et al., 2017; Miranda-Astudillo et al., 2018) which allows us to consider them as a different group inside the F-ATPases.

Finally, the latest structures of F-ATPases have shown that the membrane part of the peripheral stalk is also involved in hosting one of the half channels that define the proton path. In the chloroplast enzyme subunit *b* was found to be close to the hairpin helices of subunit *a* (Hahn et al., 2018), and in the yeast enzyme the cytoplasmic half channel was found to be formed by residues contributed by subunits *f* and *b*, in addition to the ones provided by the hairpin helices of subunit *a* (Srivastava et al., 2018). These and all of the observations described in this section suggest the peripheral stalk is a structure that contributes to the function of the enzyme way beyond acting as its stator.

## Conclusion

All the work that has been done to deepen the understanding of the peripheral stalk has consistently shown that it is an essential component of all rotary ATPases. The information obtained from highly diverse organisms, from bacteria to human and including archaea and parasites, confirms that, in spite of being a variable structure, the nature of its subunits (from their size and secondary structure to their arrangement into right-handed coiled coils), as well as their interactions and functions, are all conserved. That being said, the organisms whose ATPase has divergent features also need to be considered, since there is growing evidence suggesting that they may be the exception to the rule.

High-resolution structures of rotary ATPases have confirmed most of the previous biochemical evidence and have contributed relevant new information. These structures have not only allowed to observe the interactions of each component of the peripheral stalk, but also the different conformations in which they can be found, thus confirming both the existence and the need for flexibility in rotary ATPases, partly due to the peripheral stalk itself. The observed dynamics allowed by the nature of the subunits of the peripheral stalk and the interactions that they keep with the rest of the subunits of the enzyme have led some to think that sector R_O_ can communicate and coordinate with what happens in sector R_1_, even though they are over 100 Å apart (Stewart et al., 2013); the available evidence postulates the peripheral stalk as the main candidate for establishing such communication. In the case of F-ATPases, the detailed analysis of the structure of the peripheral stalk has shown that it is involved in processes beyond the catalytic function of the enzyme. Much of the evidence related to the dimerization and oligomerization of F-ATPases indicates that the peripheral stalk, at least the transmembrane section, is crucial for the formation and maintenance of the supramolecular associations of the enzyme and, consequently, of the peculiar mitochondrial morphology.

Based on all that has been discovered to date, it can be concluded that rotary ATPases are indeed highly dynamic enzymes, and that this characteristic is not only imposed by the mobile elements but also by those that had initially been considered static, as is the case of the peripheral stalk. Finally, and especially considering the latest studies, it can be said that the observed distortions of the enzyme involve the contribution of individual subunits, from the peripheral stalk and other parts, and illustrate the fine orchestration that this rotary enzyme is capable of building up in order to reach its maximal efficiency.

## Author Contributions

LC-T and DG-H conceptualized and wrote the original draft. LC-T, AD, HM-A, and M-FG contributed in formal analysis. AD, HM-A, and M-FG reviewed and edited the manuscript. MF-G and DG-H contributed in project administration and funding acquisition.

## Conflict of Interest Statement

The authors declare that the research was conducted in the absence of any commercial or financial relationships that could be construed as a potential conflict of interest.
